# The Roles of the Kisspeptin System in the Reproductive Physiology of the Lined Seahorse (*Hippocampus erectus*), an Ovoviviparous Fish With Male Pregnancy

**DOI:** 10.3389/fnins.2018.00940

**Published:** 2018-12-11

**Authors:** Huixian Zhang, Bo Zhang, Geng Qin, Shuisheng Li, Qiang Lin

**Affiliations:** ^1^CAS Key Laboratory of Tropical Marine Bio-Resources and Ecology (LMB), Guangdong Provincial Key Laboratory of Applied Marine Biology (LAMB), South China Sea Institute of Oceanology, Institution of South China Sea Ecology and Environmental Engineering, Chinese Academy of Sciences, Guangzhou, China; ^2^University of Chinese Academy of Sciences, Beijing, China; ^3^State Key Laboratory of Biocontrol, School of Life Sciences, Sun Yat-sen University, Guangzhou, China

**Keywords:** kisspeptin, GPR54, seahorse, male pregnancy, ovoviviparity

## Abstract

The kisspeptin/GPR54 system plays a crucial role in the regulation of the reproductive axis in vertebrates. Male pregnancy and ovoviviparity are special reproductive phenomena among vertebrates. To better understand the neuroendocrine mechanisms of male pregnancy, cDNAs encoding *kiss2* and GPR54 were cloned and functionally characterized from the lined seahorse, *Hippocampus erectus*, an ovoviviparous teleost with male pregnancy. The core mature peptide of seahorse Kiss2 is high conserved among seahorses, but unique among vertebrate Kiss orthologs. In the phylogenic analysis, the seahorse Kiss clustered with the teleost Kiss2 clade. The *kiss2* transcripts were shown to be widely expressed in various tissues, notably in the brain and gonad of the seahorse, while GPR54-2 mRNA was expressed exclusively in the brain. In addition, *kiss2* mRNA found in male seahorse brain tissue increased significantly at the early pubertal stage, and decreased significantly during pregnancy. Intraperitoneal administration of seahorse Kiss2-10 to sexual mature male seahorses demonstrated to stimulate lutropin β (LHβ) and follitropin β (FSHβ) release and increased serum testosterone levels. In summary, we first identified the kisspeptin/GPR54 system in an ovoviviparous fish with male pregnancy, which might be involved in the regulation of the reproductive functions of pubertal onset, gonadal development, and male pregnancy via regulating the synthesis of both gonadotropic hormone (GTH) and testosterone.

## Introduction

Kisspeptin is a novel neuropeptide product encoded by the *kiss* gene, which is now recognized as a key regulator of reproduction and involved in pubertal onset and neuroendocrine control of fertility in mammals (Tena-Sempere, 2010). Kisspeptin is a member of the hypothalamic RFamide family, as it possesses the Arg–Phe-amide motif at the C-terminal of the prepropeptide (Ukena and Tsutsui, 2005). Kisspeptin was first called metastin in consideration of its suppressive effects on tumor growth and tumor metastasis (Lee et al., 1996). The receptor for kisspeptin was previously characterized as an orphan G protein-coupled receptor, GPR54 (Ohtaki et al., 2001). In late 2003, a mutation of GPR54 was found in an individual with hypogonadism, and caused the absence of puberty, indicating that the kisspeptin system plays a role in the regulation of reproductive function (Roux et al., 2003).

Many studies related to the kisspeptin system in vertebrates have been reported in the last decade. In most mammals, there is only one kisspeptin gene (*Kiss1*). However, the platypus, a mammalian monotreme, has two forms of kisspeptin genes (*Kiss1* and *Kiss2*) (Lee et al., 2009). In teleosts, multiple forms of kisspeptin genes have been identified. Most teleost fishes have two paralogous kisspeptin genes, named *kiss1* and *kiss2*. However, some fish species, including green puffer, tiger puffer, stickleback, and orange-spotted grouper, lack the *kiss1* gene and possess only *kiss2* (Tena-Sempere et al., 2012; Mechaly et al., 2013), whereas *Xenopus* carries three forms of kisspeptin gene, *kiss1a*, *kiss1b*, and *kiss2* (Moon et al., 2009).

The presence of two kisspeptin gene isoforms points to the concurrent existence of multiple forms of receptor. Multiple forms of GPR54 (GPR54-1 and GPR54-2) have been characterized in many vertebrate species, including zebrafish, medaka, goldfish, striped bass, sea bass, and chub mackerel. However, only GPR54-2 was identified in tiger puffer, green puffer, gray mullet, fathered minnow, cobia, Nile tilapia, orange-spotted grouper, and half-smooth tongue sole (Tena-Sempere et al., 2012; Wang et al., 2017). In addition, *Xenopus* and European eel possess three receptor isoforms, GPR54-1a, GPR54-1b, and GPR54-2 (Moon et al., 2009; Pasquier et al., 2012a).

In mammals, many studies strongly suggest that the kisspeptin system regulates pubertal onset through the stimulation of gonadotropin-releasing hormone (GnRH) secretion. Mutations in the kiss receptor gene are related to an absence of pubertal onset and hypogonadism in human (Roux et al., 2003; Seminara et al., 2003). This abnormality was due to the disruption of the hypothalamic–pituitary–gonadal (HPG) axis, especially the kisspeptin-gonadotropin-releasing hormone (GnRH)–luteinizing hormone (LH) pathway. The GPR54 transcripts are colocalized with GnRH neurons, indicating that kisspeptin can directly stimulate gonadotropin-releasing hormone via GPR54 (Messager et al., 2005).

In teleosts, there is debate on whether fish kisspeptin is an important regulator of the reproductive HPG axis or not. Many studies suggest that fish kisspeptin, similar to mammalian kisspeptin, is a key activator of the reproductive axis. In cichlid fish (e.g., *Oreochromis niloticus* and *Astatotilapia burtoni*), striped seabass, and chub mackerel, the kisspeptin receptor mRNA was expressed in GnRH1 neurons (Parhar et al., 2004; Ohga et al., 2017). Furthermore, the injection of Kiss2 peptide promotes the expression of gonadotropins (LHβ and FSHβ) both at the mRNA and plasma levels in several fish species (Li et al., 2009; Chang et al., 2012; Kim et al., 2014; Ohga et al., 2014). However, in zebrafish and medaka, kisspeptin and GPR54 knockout experiments showed that the kisspeptin system was dispensable for reproduction (Tang et al., 2015; Nakajo et al., 2018). In addition, GnRH neurons do not express kisspeptin receptor genes in medaka (Kanda et al., 2013) or European sea bass (Escobar et al., 2013). Taken together, the role of kisspeptin in fish reproduction remains controversial, and seems to differ depending on species.

The lined seahorse (*Hippocampus erectus*) is a small marine fish with a special morphology, belonging to the order Syngnathiformes and the family Syngnathidae (fused jaws). The seahorse is an ovoviviparous fish and has a unique reproduction system with male pregnancy (Scobell and MacKenzie, 2011). The female seahorse deposits eggs into the male seahorse’s special organ called brood pouch, and the eggs get fertilized in the brood pouch. The male seahorse incubates developing embryos in the brood pouch, similar to the mammalian placenta, in which they are aerated, osmoregulated, protected, and provisioned before the hatching stage (Stolting and Wilson, 2007). The lumen of the brood pouch is rich in blood vessels and surrounded by loose connective tissue, named pseudo-placenta. The embryos are embedded within individual compartments formed from the pseudo-placenta (Dzyuba et al., 2006). However, few studies have examined the underlying hormonal mechanisms that mediate the special reproductive behavior and male pregnancy in seahorses.

Our team has recently sequenced the whole genome of the lined seahorse (Lin et al., 2017). This helped in obtaining a series of genes involved in the endocrinology regulation of reproduction. To elucidate the roles of the kisspeptin system in reproduction and male pregnancy in seahorse, the kisspeptin gene was isolated from the lined seahorse, and the expression of the gene was analyzed in a variety of tissues in male and female seahorses, as well as in the brain and testis at different gonad developmental stages, or after treatment with Kiss2-10 injection.

## Materials and Methods

### Animals and Tissue Sampling

The experimental lined seahorse individuals were collected from the Shenzhen Seahorse Center of the South China Sea Institute of Oceanology, Chinese Academy of Sciences (SCSIO-CAS), and animal ethics approval for experimentation was granted by the Chinese Academy of Sciences. The seahorses were maintained in recirculating holding tanks with seawater at room temperature under a cyclical light/dark photoperiod (16 h: 8 h). The seahorses were fed twice daily with frozen *Mysis* spp. The temperature, salinity, pH, light intensity, and dissolved oxygen (DO) were maintained at (mean ± SD) 22 ± 0.5°C, 25 ± 1.0‰, 7.9 ± 0.4, 2000 lx, and 6.5 ± 0.5 mg L^-1^, respectively.

### Cloning of the Seahorse Kisspeptin System

Adult seahorses were anesthetized with 0.05% MS222 (Sigma, Shanghai, China). Total RNA was extracted from the brain using TRIzol reagent (Invitrogen, CA, United States) according to the manufacturer’s instructions. First-strand cDNA was synthesized using the PrimeScript^TM^ RT Reagent Kit with gDNA Eraser (Takara, Dalian, China). Partial cDNA fragments were obtained by PCR using primers (Table 1) designed based on the sequences of *kiss2* and GPR54-2 from the published genome data of lined seahorse (Lin et al., 2017). To obtain the full-length sequence of the cDNA of the kisspeptin gene in lined seahorse, 3′ and 5′ rapid amplification of cDNA ends (RACE) PCRs were conducted using the SMARTer RACE Kit (Clontech, CA, United States) according to the manufacturer’s protocols. PCR amplification was performed using Taq DNA Polymerase Mix (TAKARA, Dalian, China), and PCR conditions were as follows: denaturation at 94°C for 5 min, followed by 35 cycles at 94°C for 30 s, 56°C for 30 s, 72°C for 2 min, and a final incubation at 72°C for 10 min. The PCR products were purified from an agarose gel and subcloned into a pGEM-T Easy Vector System (Promega, Fitchburg, WI, United States). The inserts were sequenced using an ABI 3700 sequencer (Applied Biosystems).

**Table 1:** Information of primers used in this study.

Gene	Purpose	Primer	5′–3′ sequence
**Primer sequence**			
kiss2	Partial cDNA	kiss2F1	CTCCCAAGATGAAGTT TGCAG
		kiss2R1	TCAGGTCAGCACCTC CAGTTG
	5′RACE	kiss2R2 (first)	AGATGGACGCATG ATTGTAG
		kiss2R3 (nest)	TTTGTTCCTGTGG TCGTTGC
	3′RACE	kiss2F2 (first)	CCTGAGCAGGAAT CACAGGG
		kiss2F3 (nest)	ACCTTTGTTTCTCC CTGATAG
	Real-time PCR	kiss2qF	ATCCCAACCTTTGT TTCTCC
		kiss2qR	AAATCTGCTTGTT CTGGCTCT
GPR54-2	Partial cDNA	GPR54-2F1	GCAGCATCCCTTTCT TACCGA
		GPR54-2 R1	GACCTGGTAGTTGT TGTCTCC
	5′RACE	GPR54-2 R2 (first)	TGGTAGAGGATGAAG GCCCTC
		GPR54-2 R3 (nest)	CACCTGCTGCAAGAAG GCCAC
	3′RACE	GPR54-2 F2 (first)	AGGTCTCCAAGATG GTGGTCG
		GPR54-2 F3 (nest)	GATCAAGACGTGGGC AAACTG
	Real-time PCR	GPR54-2qF	AGCCAGGAGACAACA ACTACC
		GPR54-2qR	GAGGAGTTGGCATAA GACATG
sGnRH	Real-time PCR	sGnRHqF	CCTTGCGTAGCTGAGA TGGAG
		sGnRHqR	TACATTGTATGGTC GACGTCTC
LHβ	Real-time PCR	LHβqF	CACAAGGAACCCAC TAAACC
		LHβqR	GAGGGTGCTTTCTT TATTCTG
FSHβ	Real-time PCR	FSHβqF	GCAATGGGAACTGGA CCTAC
		FSHβqR	TGATTGATACGA GCAGCACA
β-actin	Real-time PCR	β-actin_qF	TTCACCACCACAG CCGAGA
		β-actin_qR	TGGTCTCGTGGA TTCCGCAG


### Structural, Phylogenetic and Syntenic Analyses of Seahorse Kisspeptin System

The amino acid sequences of *kiss2* and GPR54-2 in lined seahorse were translated from the nucleotide sequence using DNASTAR software. The putative signal peptides were predicted by SignalP 4.1 ^1^. The putative transmembrane domain of GPR54-2 was predicted using the TMHMM server V2.0 ^2^. Multiple sequence alignments of amino acids were performed with ClustalX 2.0 (Larkin et al., 2007). Protein phylogeny analyses were conducted with MEGA6.0 (Tamura et al., 2013) using the neighbor-joining method with 1000 bootstrap replicates. TBLASTN searches against the genome assemblies of different vertebrate species, including human, zebrafish, medaka, and stickleback, and data retrieval for synteny analysis were performed using the Ensembl genome browser^3^.

### Tissue Distribution of the Seahorse Kisspeptin System

To detect the tissue distribution of *kiss2* and GPR54-2 mRNA in lined seahorse, three adult female (11.3 ± 0.26 g, mean body weight) (8 months old) and male lined seahorses (12.7 ± 0.45 g) (8 months old) were anesthetized with 0.05% MS222, and decapitated. Tissue samples of the brain, gill, liver, intestine, kidney, muscle, brood pouch, skin, and gonads of male and female seahorses were quickly collected, snap frozen in liquid nitrogen, and stored at -80°C until RNA extraction. Different brain regions including telencephalon, cerebellum, optic tectum-thalamus, hypothalamus and pituitary were also detected. One micro gram of total RNA from each tissue was digested with a genome eraser and reverse-transcribed into cDNA using the PrimeScript^TM^ RT Reagent Kit with gDNA Eraser (Takara, Dalian, China).

### Expression Profile of *kiss2* During Sexual Development Stages

The expression profile of seahorse *kiss2* was analyzed in the whole brain and testis of four different sexual development stages including immature (IMM), early puberty (EP), advanced puberty (AP), and mature (MAT) (3, 4, 5, and 6 months old) (*n* = 8) by real-time PCR. The gonad tissues of seahorses in different stages were fixed in Bouin’s solution for 24 h. Then, the gonads were embedded in paraffin, cut into 10 μm sections, and stained with hematoxylin and eosin. The classification of the gonad development stages was determined by light microscopy.

### Expression Profile of *kiss2* During the Pregnancy Stages

Adult seahorses were allowed to mate freely before being subjected to a standardized assessment of pregnancy status on the basis of courtship behaviors. The pregnant seahorses (P stage) (*n* = 8) were maintained in the tank before euthanasia to sample the brain and testis tissues. Adult seahorses that were already mature were termed the pre-pregnancy group (PreP stage) (*n* = 8), and post-parturition (PostP stage) seahorses, in which the larval seahorses had hatched, were termed the post-pregnancy group (PostP stage) (*n* = 8).

### Administration of Kisspeptin to Seahorse

Synthetic peptides corresponding to the lined seahorse Kiss2 decapeptide (Kiss2-10) (FNVNPFGLRF-NH2) were provided by China Peptide Co., Ltd. (Shanghai, China) with a purity of 99.75%, as determined by HPLC. To evaluate the role of *kiss2* in the hypothalamus–pituitary axis, male lined seahorses were injected with Kiss2-10. The mRNA expression levels of GPR54 and sGnRH in the hypothalamus and FSHβ and LHβ in the pituitary were detected by real-time PCR. Sexually mature male seahorses, 12.5–14.0 g in body weight, were kept in indoor tanks supplied with recirculating sea water and feed with frozen *Mysis* spp. The test Kiss2-10 peptides were dissolved in a vehicle of 0.7% NaCl. Seahorses were anesthetized with 0.05% MS222 and intraperitoneally injected with Kiss2-10 (20 nmol/g body weight at a volume of 20 μL/g body weight) and saline (20 μL/g body weight) as a negative control. Eight seahorses from each group were collected randomly and killed by decapitation at 3 and 6 h post-injection. The hypothalamus and pituitaries were quickly dissected, frozen in liquid nitrogen, and stored at -80°C for subsequent RNA extraction. Blood samples were collected from tail vessels at 3 and 6 h post-injection. Serum samples were separated by centrifugation at 2,500 ×*g* for 30 min at 4°C, and stored at -20°C until the measurement of testosterone by ELISA as described in the manufacturer’s protocol (Cayman Chemical Company, Ann Arbor, MI, United States).

### RNA Extraction, Reverse-Transcription, and Real-Time Quantitative PCR

All experiments were performed according to the standard procedure in our lab (Zhang et al., 2016; Qin et al., 2018). In brief, total RNA was extracted using TRIzol reagent (Invitrogen, United States) according to the manufacturer’s protocol. The purity and yield of RNA were detected by a NanoDrop 2000C spectrophotometer (Thermo Fisher Scientific, United States), and the integrity of RNA was determined by gel electrophoresis. One microgram of RNA was used as a template for the first strand cDNA synthesis using the reverse-transcription kit described above. Real-time quantitative PCR (qPCR) was performed on a Roche Light-Cycler 480 real time PCR system using SYBR Premix Ex Taq^TM^ (TAKARA, Japan). qPCR conditions were as follows: denaturation at 94°C for 3 min, followed by 40 cycles at 94°C for 15 s, 55–58°C for 15 s, and 72°C for 20 s. At the end of the amplification, a melting curve analysis was generated to confirm the presence of a single PCR product. The housekeeping gene β-actin was used as an internal reference gene. The expression levels of each target gene analyzed via qPCR were determined using the comparative quantification method 2^-ΔΔCT^ (Livak and Schmittgen, 2001).

### Statistical Analyses

All the data were expressed as the means ± standard error of mean (SEM) and evaluated by one way analysis of variance (ANOVA) followed by Duncan’s multiple-range tests using Prism6.0 (GraphPad software, CA, United States) and SPSS 19.0 (IBM, United States). Differences between groups with *p* < 0.05 were considered statistically significant.

## Results

### Cloning and Characterization of *kiss2* and GPR54-2 in Seahorse

**FIGURE 1:**
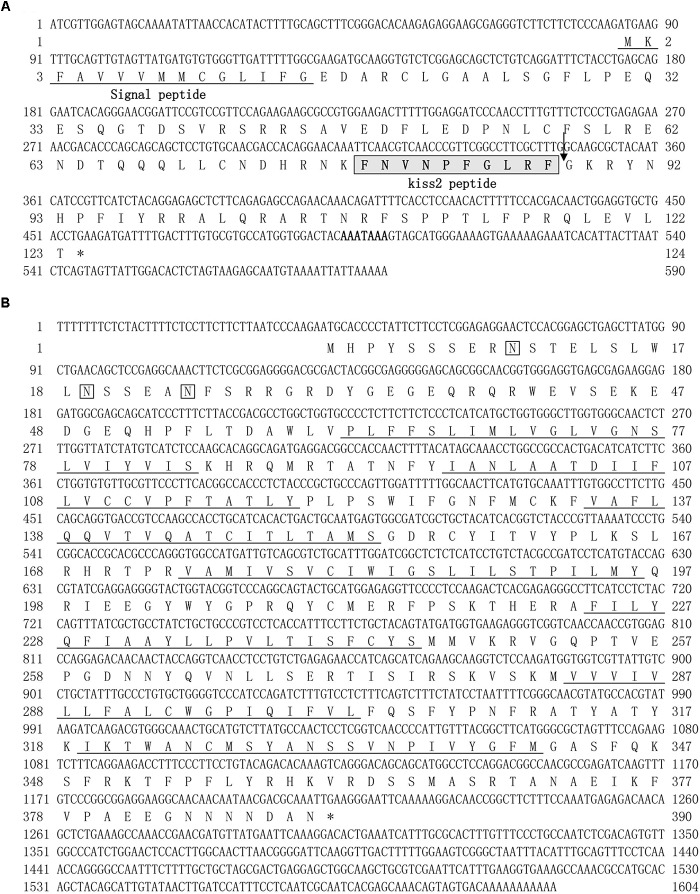
cDNA and deduced amino acid sequence of lined seahorse *kiss2 and* GPR54-2. **(A)** The nucleotide and deduced protein sequences of *kiss2* cDNA. The putative signal peptide is underlined. The mature peptide (kisspeptin-10) is boxed. **(B)** The nucleotide sequence and deduced protein sequence of GPR54-2 cDNA in lined seahorse. Seven putative hydrophobic transmembrane (TM) domains are underlined. Putative N-linked glycosylation sites are indicated by diamonds (CBS prediction server score > 0.50).

**FIGURE 2:**
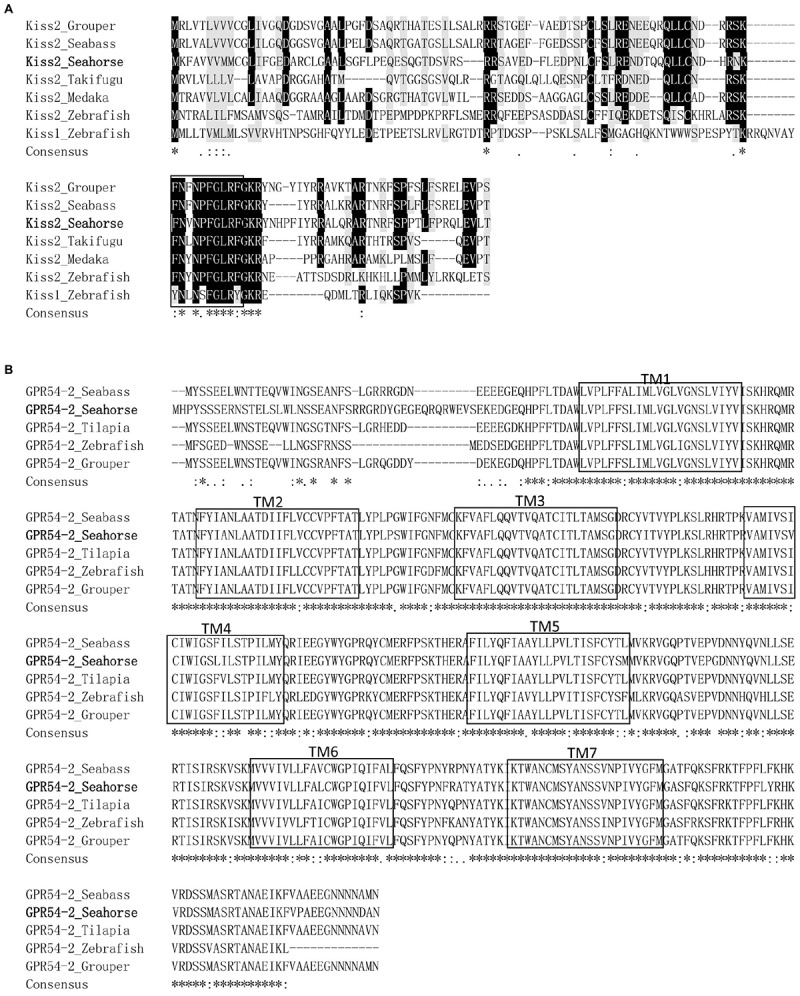
Comparison of amino acid sequences of kisspeptin precursors and GPR54-2 from different species. **(A)** The mature peptides of Kiss2 are boxed. Multiple sequence alignment was performed by ClustalX 2.0. Identical sequences are indicated by asterisks. **(B)** Alignment of the amino acid sequences of GPR54-2 in different species. Gaps introduced in some sequences to maximize the alignment are indicated by hyphens. Identical sequences are indicated by asterisks. Putative TM domains are boxed. The GPR54-2 sequences of other teleosts were downloaded from GenBank (For detailed, see the legend for Figure 3).

**FIGURE 3:**
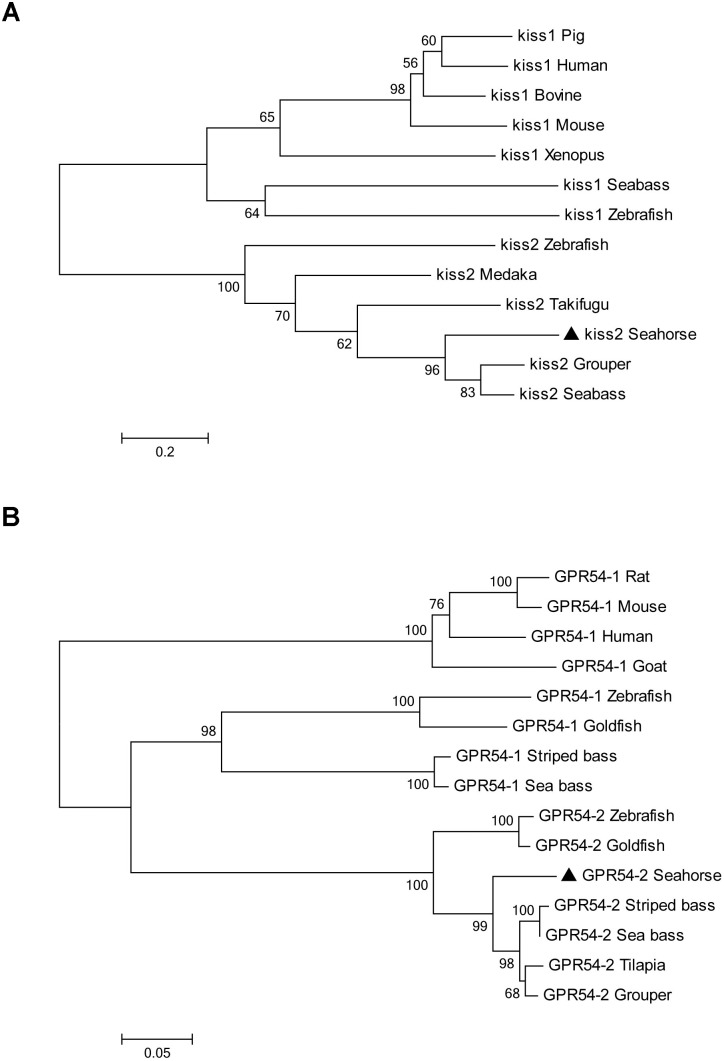
Phylogenetic tree depicting the evolutionary relationships of lined seahorse Kiss2 and GPR54-2 with those of other vertebrates. The phylogenetic tree was contrasted by MEGA6.0 using the neighbor-joining method with 1000 bootstrap replicates. **(A)** The tree was constructed from the deduced amino acid sequences of vertebrate Kiss listed in Table 2. Numbers at nodes indicate the bootstrap value (%), obtained for 1000 replicates. Kiss GenBank Accession Nos. are listed in Table 2. **(B)** Phylogenetic tree of GPR54 in vertebrates. The number shown at each branch indicates the bootstrap value (%). GenBank accession numbers are as follows: human (*Homo sapiens*) GPR54-1 (NP_115940); rat (*Rattus norvegicus*) GPR54-1 (NP_076482); mouse (*Mus musculus*) GPR54-1 (NP_444474); goat (*Capra hircus*) GPR54-1 (ACY91951); zebrafish (*Danio rerio*) GPR54-1 (ABV44613), GPR54-2 (ABV44612); goldfish (*Carassius auratus*) GPR54-1 (ACK77793), GPR54-2 (ACK77792); striped bass (*Morone saxatilis*) GPR54-1 (AID62214), GPR54-2 (ADU54205); European sea bass (*Dicentrarchus labrax*) GPR54-1 (AFK84355), GPR54-2 (AFK84356); orange-spotted grouper (*Epinephelus coioides*) GPR54-2 (ACT65994); Nile tilapia (*Oreochromis niloticus*) GPR54-2 (BAD34454); and lined seahorse (*Hippocampus erectus*) GPR54-2 (MH514014).

**FIGURE 4:**
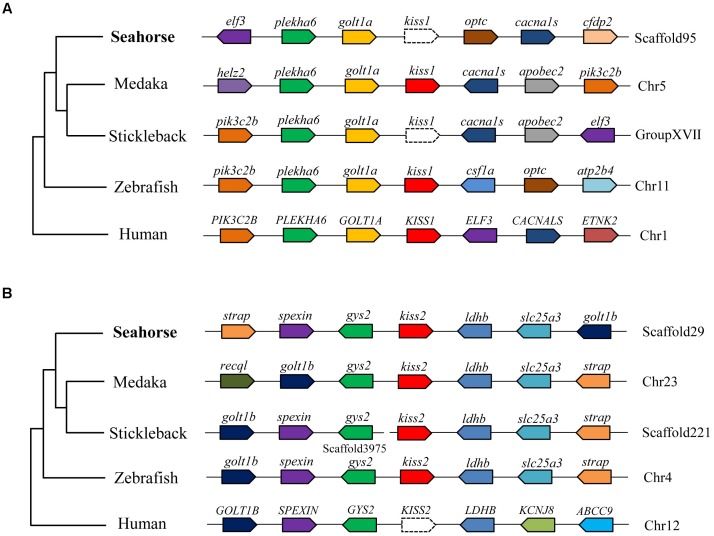
Conserved synteny for the genomic region comprising *kiss1* **(A)** and *kiss2* **(B)** genes. Gene loci organizations in the genomic region containing the kisspeptin gene were obtained from the Ensembl genome browser (http://www.ensembl.org).

A full-length cDNA encoding the *kiss2* precursor was isolated from the lined seahorse hypothalamus (GenBank Accession Number: MH514013). The open reading frame (ORF) encodes for the 124 amino acid (aa) precursor Kiss2 protein, with an N-terminal putative signal peptide sequence of 15 aa (Figure 1A). Sequence alignment of deduced protein sequences shows that seahorse and other vertebrate Kiss precursors have relatively low identities with each other (Table 2). Homology analysis revealed that seahorse Kiss2 had the highest sequence identities with European seabass (62.2%) and orange-spotted grouper (58.2%) among Perciformes species. However, the mature decapeptide of Kiss (Kiss-10) and the C-terminal cleavage site (GKR) are conserved (Figure 2A). Interestingly, the decapeptide of seahorse Kiss2 (FNVNPFGLRF) is very unique. Phylogenetic analysis shows that Kiss cDNA sequences are clustered into two separate clades: *kiss1* and *kiss2*. The seahorse *kiss* is clustered with the *kiss2* clade and shares the highest similarity with seabass and grouper *kiss2* (Figure 3A). The synteny analysis of *kiss* shows that the *kiss2* gene is usually positioned in the genomic regions, including *golt1b*, *spexin*, *gys2*, and *ldhb*. The position of *kiss2* in seahorse and other teleost fishes is conserved (Figure 4B). The *kiss1* gene is usually positioned in the genomic regions containing common loci, including *plekha6*, *golt1a*, and *elf3*. While in this loci, the *kiss1* gene of seahorse is absent (Figure 4A).

**Table 2:** Amino acid sequence identities of the lined seahorse *kiss2* with *kiss* genes of various vertebrates.

Species	Accession no.	Amino acid sequence identities of Kiss (%)
		Seahorse Kiss2
**Identity of vertebrate Kiss protein**		
Human Kiss1	NP002247	10.6
Pig Kiss1	ACH68409.1	9.8
Bovine Kiss1	XM867473.1	13.2
Mouse Kiss1	AAI17047	13.8
Xenopus Kiss1	BX850386	13.7
Seabass Kiss1	FJ008914	8.2
Zebrafish Kiss1	ABV03802	12.8
Takifugu Kiss2	BAJ15497	47.1
Grouper Kiss2	ACT65993	58.2
Medaka Kiss2	AB439562	43.0
Seabass Kiss2	FJ008915	62.2
Zebrafish Kiss2	AB43956.1	26.9


The cloned full-length *GPR54-2* cDNA sequence is 1604 bp (GenBank number: MH514014), including an ORF of 1173 bp, 39 bp of 5′UTR and 392 bp of 3′UTR. The deduced 390 amino acids contain an extracellular N-terminus, a seven transmembrane domain and a cytoplasmic C-terminus. Three potential N-linked glycosylation sites were predicted in the extracellular N-terminus (Figure 1B). Multiple amino acid sequence alignments of *GPR54* showed that the identity of *GPR54* from different teleost species is very high except the extracellular N-termius domain (Figure 2B). In the phylogenetic analysis, the seahorse *GPR54* clusters into *GPR54-2* clade (Figure 3B).

### Tissue Distribution of *kiss2* and GPR54-2 in Seahorse

**FIGURE 5:**
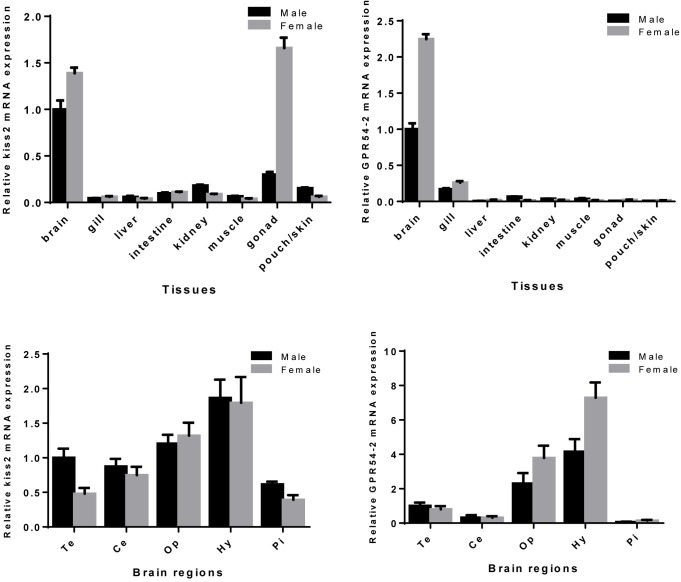
Relative mRNA expression levels of *kiss2* and GPR54-2 mRNA in various tissues and brain regions of male and female seahorses. The brain regions including: Te, telencephalon; Ce, cerebellum; Op, optic tectum-thalamus; Hy, hypothalamus; Pi, pituitary. The mRNA expression levels are identified by real-time PCR, normalized against the β-actin transcript, and presented as means ± SEM.

Expression patterns of seahorse *kiss2* and GPR54-2 in various tissues and brain regions were examined by real-time PCR. The *kiss2* mRNA was expressed in all detected tissues and was highly expressed in the brain and ovary (Figure 5 and Supplementary Figure S1). Interestingly, *kiss2* mRNA was expressed in the brood pouch of male seahorse. However, GPR54-2 was expressed exclusively in the brain of seahorse, and the expression levels were close to the detection limits in the peripheral tissues. The *kiss2* and GPR54-2 mRNA were highly expressed in the hypothalamus region of brain of both male and female seahorses, compared to other brain regions. In addition, *kiss2* and GPR54-2 mRNA levels of expression in the brain tissue were found to be higher in females than in males.

### Expression Profile of *kiss2* During Sexual Development in Male Seahorse

**FIGURE 6:**
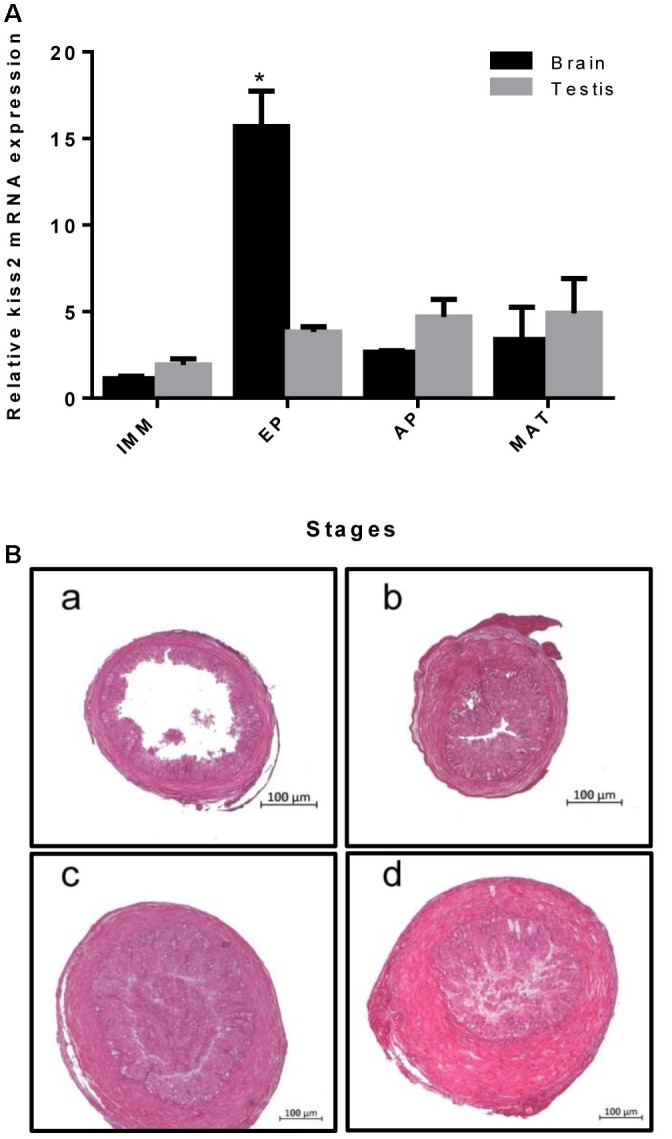
**(A)** The expression profiles of *kiss2* in the brain and testis of male lined seahorses (*n* = 8) during different gonad developmental stages. **(B)** **(a–d)** Histology of gonads at different gonadal developmental stages in male lined seahorses. **(a)** Immature (IMM) (3 months old); **(b)** Early puberty (EP) (4 months old); **(c)** Advanced puberty (AP) (5 months old); **(d)** Mature (MAT) (6 months old). The mRNA expression levels were identified by real-time PCR, normalized against the β-actin transcript, and presented as means ± SEM. Asterisks denote significant differences between different stages (*P* < 0.05).

Real-time quantitative PCR was used to quantify the expression of *kiss2* in the brain and testis of male seahorses during the gonadal developmental stages. The *kiss2* mRNA expression in the brain was significantly high in the early pubertal stage (4-month-old class) (*P* = 0.0312, *P* < 0.05), while there was no significant difference in the testis (*P* = 0.764) (Figure 6).

### Expression Profile of *kiss2* During the Pregnancy Stages

**FIGURE 7:**
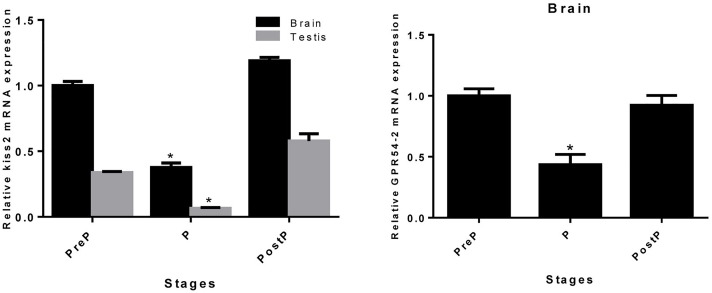
The mRNA expressions of *kiss2* in the brain and testis (*n* = 8) and GPR54-2 in the brain of the male seahorses during different pregnant stages. PreP, pre-pregnant stage; P, pregnant stage; PostP, post parturition stage. The mRNA expression levels were identified by real-time PCR normalized against the β-actin transcript and presented as means ± SEM. Asterisks denote significant differences between different stages (*P* < 0.05).

The expression pattern of seahorse *kiss2* during the pregnancy stages was detected by real-time PCR. Both in the brain and testis, the expression level of *kiss2* mRNA was reduced significantly during pregnancy (P stage), and returned to the normal levels post-parturition (brain: *P* = 0.0267, *P* < 0.05; testis: *P* = 0.0395, *P* < 0.05). The GPR54-2 mRNA was also downregulated during the pregnant stage (*P* = 0.0452, *P* < 0.05) (Figure 7).

### Effects of Administration of Kiss2-10 on the Hypothalamus-Pituitary Axis in Seahorse

**FIGURE 8:**
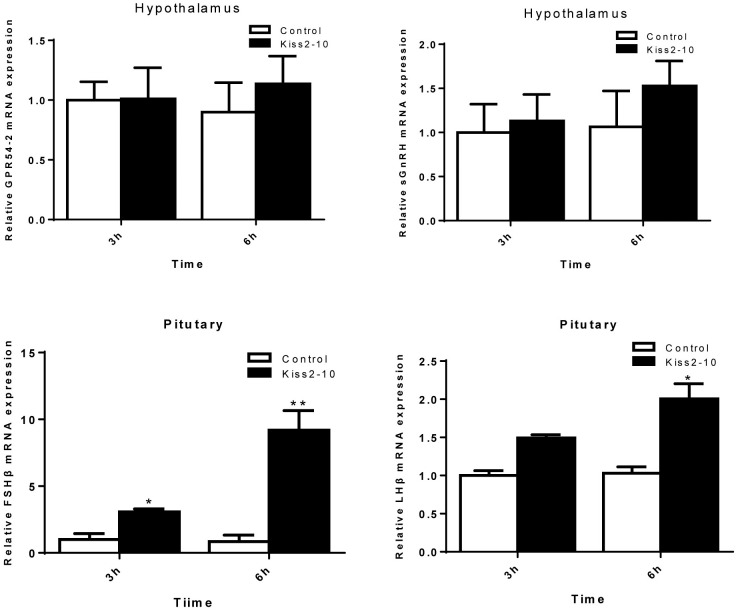
Effects of administration of seahorse Kiss2-10 on the gene expressions for GPR54-2, sGnRH, FSHβ, and LHβ at 3 and 6 h post-injection in sexually mature male lined seahorses. The mRNA expression levels were identified by real-time PCR, normalized against the β-actin transcript, and presented as means ± SEM. Asterisks denote significant differences between different treatments (*P* < 0.05).

**FIGURE 9:**
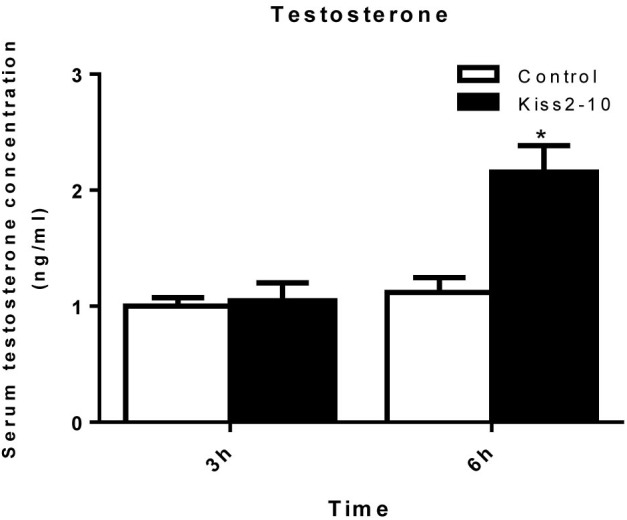
Serum testosterone concentrations post-injection of seahorse Kiss2-10. Data are presented as means ± SEM. Asterisks denote significant differences between different treatments (*P* < 0.05).

The intraperitoneal injection of Kiss2-10 significantly increased FSHβ (*P* = 0.00815, *P* < 0.01) and LHβ (*P* = 0.0297, *P* < 0.05) at 6 h post-injection. In addition, the mRNA expression of FSHβ increased significantly at 3 h post-injection (*P* = 0.0436, *P* < 0.05). However, no significant difference was found in sGnRH (*P* = 0.853) and GPR54-2 (*P* = 0.791) mRNA levels (Figure 8). The serum testosterone concentration increased significantly at 6 h post-injection (*P* = 0.0443, *P* < 0.05) (Figure 9).

## Discussion

In the present study, one kisspeptin gene and its receptor were characterized and functionally evaluated in the lined seahorse. This is the first description of the kisspeptin system in an ovoviviparous fish with male pregnancy. The deduced amino acid sequence of seahorse *kiss2* is poorly conserved compared to that of other vertebrate Kiss orthologs. However, the core putative mature peptide (decapeptide, Kiss2-10) and the GKR cleavage site were conserved. The Kiss2-10 is unique (FNVNPFGLRF) among the vertebrate kisspeptins, while the sequence of Kiss2-10 in most vertebrates is FNFNPFGLRF (Pasquier et al., 2012b). The Kiss2-10 of the lined seahorse differed by only one amino acid to the Kiss2-10 of zebrafish (FNYNPFGLRF) and differed significantly by four aa to the Kiss1-10 of zebrafish (YNLNSFGLRY). The phylogeny tree showed that the *kiss2* of seahorse clustered with the teleost fish *kiss2* genes, and the synteny of *kiss2* in seahorse is conserved with other teleost fishes. These findings demonstrated that the cloned kisspeptin gene in seahorse is the orthologous gene of *kiss2* in the vertebrates.

Based on the genome and transcriptome databases of the lined seahorse, we searched for the *kiss1* gene in the lined seahorse genome and RNA-seq sequences by using tBLASTn search tool, and no *kiss1* gene was found. The synteny analysis of *kiss1* also showed that the *kiss1* gene was lost from the conserved locus of the lined seahorse. Similar results were found in several fish species with available genome databases, such as the stickleback (*Gasterosteus aculeatus*), tiger puffer (*Takifugu rubripes*), and green puffer (*Tetraodon nigroviridis*) (Felip et al., 2009). In addition, some fish species without sequenced genomes, such as orange-spotted grouper, grass puffer, Senegalese sole and Nile tilapia, also lacked the *kiss1* gene and possessed only the *kiss2* gene (Tena-Sempere et al., 2012). Moreover, in platy fish, another ovoviviparous fish with an available genome database, we also found the *kiss2* gene on the scaffold JH556799.1, and the *kiss1* gene was absent. In addition, seahorse only possessed GPR54-2 and lacked GPR54-1, based on the genome data. Our study provides conclusive evidence for the loss of the *kiss1* system in the genome of several vertebrate species. Considering that the evolutionary rate of seahorse is very rapid (Lin et al., 2016), one of the duplicated copies (*kiss1* and GPR54-1) in seahorse appears to have been lost during evolution.

It had been indicated that kisspeptin has been determined as an important regulator of the reproductive brain–pituitary–gonad (BPG) axis in mammals and teleosts (Ohga et al., 2018). Real-time PCR analysis supported this conclusion, showing that *kiss2* transcripts were highly expressed in the brain and gonad tissues in female and male seahorses. Interestingly, *kiss2* mRNA was expressed in the brood pouch of male seahorses. The brood pouch protected the embryos and provided them with oxygen and nutrients (Stolting and Wilson, 2007). This result suggested that kisspeptin may play a role in the regulation of male pregnancy in the lined seahorse. Moreover, GPR54-2 mRNA was mainly expressed in the brain while expressed much lower levels in the peripheral tissues of the lined seahorse. This expression pattern was consistent with previous studies. The expression of GPR54 in the brain was much higher than the expression in the gonad and other peripheral tissues in most teleosts including zebrafish, fathead minnow, chub mackerel and tongue sole (Wang et al., 2017; Ohga et al., 2018). The predominant expression of GPR54-2 mRNA in the brain indicated the potential involvement of seahorse kisspeptin in neural functions of the HPG axis as a key regulator of reproduction.

Many studies have demonstrated that kisspeptin plays a critical role in pubertal onset in vertebrates (Seminara et al., 2003); (Cortes et al., 2015). However, the roles of kisspeptin in ovoviviparous fishes are still widely underexplored. In the present study, we have detected the expression profile of *kiss2* during gonadal development in male lined seahorse. It is interesting to note that *kiss2* mRNA in the brain increased significantly at the early puberty stage in male seahorses, while there was no significant difference in the testis. This result indicated that seahorse *kiss2* was involved in the onset of puberty through the brain rather than the testis. In Atlantic halibut, kisspeptin was also involved in the onset of puberty (Mechaly et al., 2010). Moreover, in male chub mackerel, *kiss2* mRNA expression increased significantly just before the onset of meiosis in the testis (Ohga et al., 2018). These results suggest the positive involvement of the kisspeptin system in the pubertal process of teleosts, as is the case for mammals.

The mRNA expressions of *kiss2* in the brain and testis of seahorses were significantly reduced at the pregnancy stage. This result indicated that kisspeptin plays a role in the regulation of pregnancy in seahorses. In humans, the circulating kisspeptin levels are low in non-pregnant females, but dramatically increase during pregnancy (Horikoshi et al., 2003). However, kisspeptin does not appear to increase during pregnancy in many non-primate species, including rodents, sheep, and horses (Scott and Brown, 2013). It has been demonstrated that kisspeptin regulates the oxytocin system during pregnancy and lactation stages; however, the physiological role of circulating kisspeptin during pregnancy remains uncertain in mammals. To our knowledge, this is the first report that the expression of kisspeptin is reduced during pregnancy in an ovoviviparous teleost (seahorse). This suggests that the physiological role of kisspeptin in pregnancy is different between ovoviviparous teleosts and mammals.

In both mammals and teleosts, kisspeptin plays a crucial role in controlling reproductive activities by increasing gonadotropin release, which is mediated by stimulating GnRH release (Popa et al., 2008; Ohga et al., 2018). However, it is not known whether kisspeptin in ovoviviparous teleosts serves the same role on the reproductive axis. In the present study, the *in vivo* administration of synthetic seahorse Kiss2-10 potently stimulated the expression of FSHβ and LHβ in the pituitary of mature male seahorses, indicating that the stimulatory action of kisspeptin on GTH release is conserved among vertebrates. On the other hand, there is no stimulatory effect on the expression of GnRH in the hypothalamus, suggesting that Kiss2-10 does not act through GnRH releasing in seahorse, and can stimulate GTH expression directly. Moreover, seahorse Kiss2-10 injection can also stimulate the serum testosterone concentration at 6 h post-injection. In mammals, administration of kisspeptin potently increased plasma LH and testosterone (Patterson et al., 2006). These observations indicated that kisspeptin may mediate its effects on the HPG axis by increasing the gonadotropin hormone and testosterone concentration in seahorses, serving the same role as that of its mammalian counterparts on the reproductive axis. In male fishes, FSH and LH can stimulate testicular Leydig cells to produce androgens including testosterone (T) and 11-ketotestosterone (11-KT). These androgens in turn promote spermatogenesis and development of secondary sexual characteristics in males (Knapp and Carlisle, 2011). In seahorses, the Leydig cells underwent a distant seasonal cycle of activation. A prolonged period of androgen synthesis that was maximal during proliferation of spermatocytes and development of the brood pouch, but suppressed during the pregnant stage (Scobell and MacKenzie, 2011). Our findings show that seahorse *kiss2* mRNA expression was upregulated during early puberty while it appeared to be downregulated during pregnancy. And the expression profiles of kiss2 in early puberty and pregnant stages were constant with the androgen levels during these stages. This suggested that the seahorse kisspeptin levels are regulated to control the androgen level in the Leydig cells through stimulating FSH and LH.

In conclusion, we cloned *kiss2* and GPR54-2 cDNA sequences from the lined seahorse and investigated their expression profiles in various tissues and different reproductive stages. The putative mature peptide and protein cleavage sites of *kiss2* were well conserved among vertebrates. Tissue distribution showed that *kiss2* transcripts were highly expressed in the brain and gonad tissues in both female and male seahorses. However, GPR54-2 mRNA was exclusively expressed in the brain. In addition, the *kiss2* mRNA in the brain increased significantly at the early puberty stage of the male seahorse, and was reduced significantly at the pregnancy stage. We have demonstrated that seahorse Kiss2-10 stimulated LH and FSH release *in* *vivo*, and increased the serum testosterone concentration. Our results suggest that the kisspeptin/GPR54 system may be involved in the regulation of reproductive function of pubertal onset and gonadal development, as well as male pregnancy, by regulating both GTH and testosterone synthesis in the lined seahorse.

## Author Contributions

HZ and QL designed the research. HZ and BZ carried out the experiments. HZ, GQ, and SL analyzed the experiment data. QL provided lab space and equipment. All authors wrote the paper.

## Conflict of Interest Statement

The authors declare that the research was conducted in the absence of any commercial or financial relationships that could be construed as a potential conflict of interest. The handling Editor and reviewer LE declared their involvement as co-editors in the Research Topic.

## Footnotes

1 www.cbs.dtu.dk/services/SignalP/

2 www.cbs.dtu.dk/services/TMHMM/

3 http://www.ensembl.org

## Supplementary Material

Figure S1 RT-PCR analysis of tissue expression patterns of *kiss2* and GPR54-2 in male and female seahorses. Amplification of β-actin was used as house-keeping gene control. Br, brain; Li, liver; Gi, gill; In, intestine; Ki, kidney; Mu, muscle; Po, pouch; Sk, skin; Go, gonad; NC, negative control.
