# Supplementary figures and images for: The Roles of the Kisspeptin System in the Reproductive Physiology of the Lined Seahorse (Hippocampus erectus), an Ovoviviparous Fish With Male Pregnancy
Source: Front Neurosci. 2018 Dec 11;12:940. doi: 10.3389/fnins.2018.00940 (PMC6298243; doi:10.3389/fnins.2018.00940)

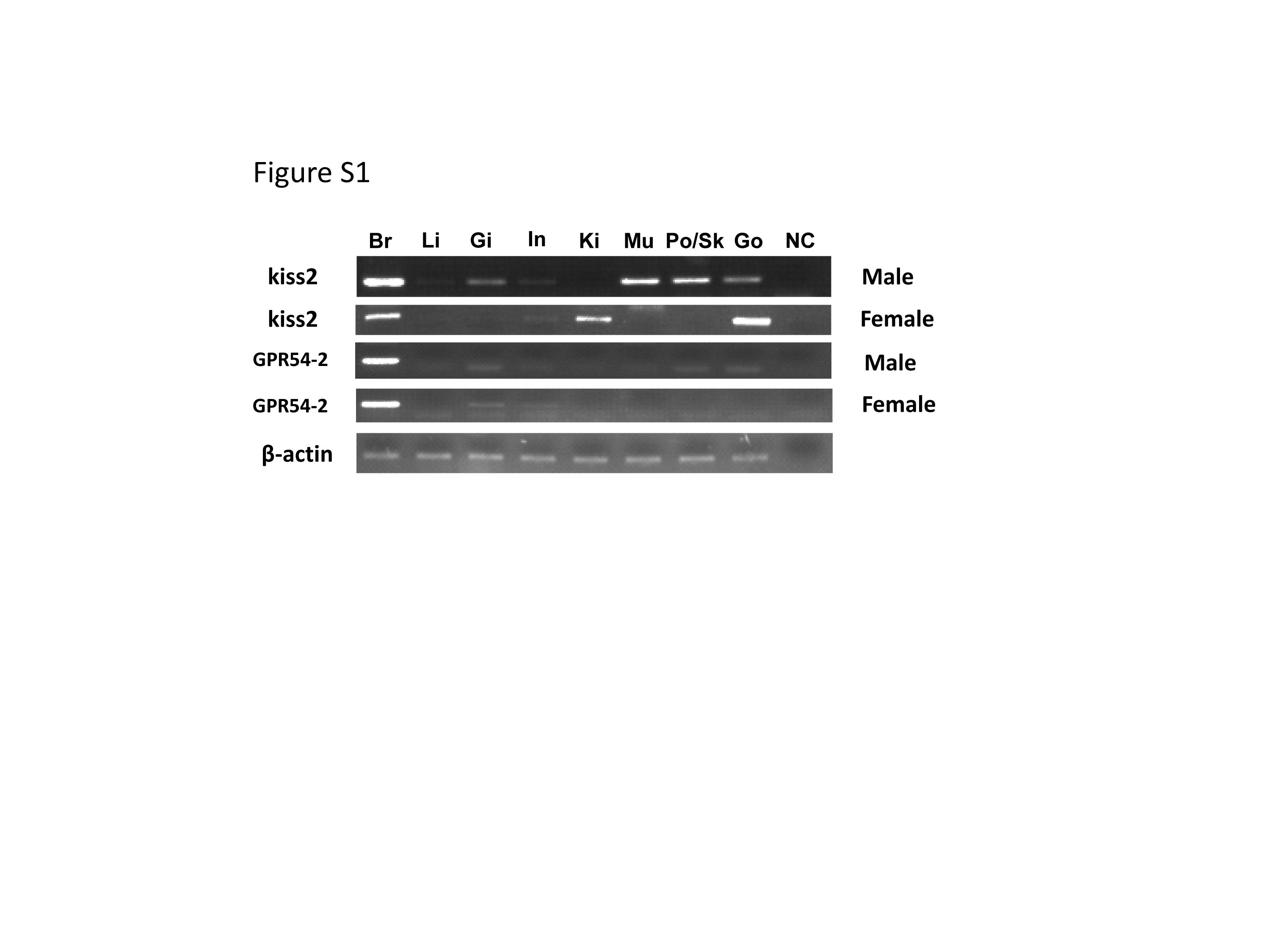

Supplement: Figure S1 — RT-PCR analysis of tissue expression patterns of kiss2 and GPR54-2 in male and female seahorses. Amplification of β-actin was used as house-keeping gene control. Br, brain; Li, liver; Gi, gill; In, intestine; Ki, kidney; Mu, muscle; Po, pouch; Sk, skin; Go, gonad; NC, negative control. [file Image_1.JPEG]
